# The dichotomy of pathogens and allergens in vaccination approaches

**DOI:** 10.3389/fmicb.2014.00365

**Published:** 2014-07-16

**Authors:** Fiona J. Baird, Andreas L. Lopata

**Affiliations:** ^1^Centre for Biodiscovery & Molecular Development of Therapeutics, Centre for Biosecurity in Tropical Infectious Diseases, Australian Institute of Tropical Health & Medicine, James Cook UniversityTownsville, QLD, Australia; ^2^Molecular Immunology Group, School of Pharmacy and Molecular Biology, James Cook UniversityTownsville, QLD, Australia

**Keywords:** vaccine, vaccine history, allergy, food allergy, allergen, prophylactic vaccination, vaccine challenges

## Abstract

Traditional prophylactic vaccination to prevent illness is the primary objective of many research activities worldwide. The golden age of vaccination began with an approach called variolation in ancient China and the evolution of vaccines still continues today with modern developments such as the production of Gardasil^TM^ against HPV and cervical cancer. The historical aspect of how different forms of vaccination have changed the face of medicine and communities is important as it dictates our future approaches on both a local and global scale. From the eradication of smallpox to the use of an experimental vaccine to save a species, this review will explore these successes in infectious disease vaccination and also discuss a few significant failures which have hampered our efforts to eradicate certain diseases. The second part of the review will explore designing a prophylactic vaccine for the growing global health concern that is allergy. Allergies are an emerging global health burden. Of particular concern is the rise of food allergies in developed countries where 1 in 10 children is currently affected. The formation of an allergic response results from the recognition of a foreign component by our immune system that is usually encountered on a regular basis. This may be a dust-mite or a prawn but this inappropriate immune response can result in a life-time of food avoidance and lifestyle restrictions. These foreign components are very similar to antigens derived from infectious pathogens. The question arises: should the allergy community be focussing on protective measures rather than ongoing therapeutic interventions to deal with these chronic inflammatory conditions? We will explore the difficulties and benefits of prophylactic vaccination against various allergens by means of genetic technology that will dictate how vaccination against allergens could be utilized in the near future.

## INTRODUCTION

Globally, the burden of disease and infection is diverse and inescapable. It is a shared aﬄiction for humanity and one that is constantly moderated by better hygiene, enhanced education, and improved vaccines and therapeutic interventions. In terms of healthcare, it is always more beneficial to prevent a disease or infection from occurring than to treat and cure it. The development of vaccines is dependent on the knowledge of: what pathogen causes the disease; how it establishes itself in the host; how the host’s innate and cell-mediated immunity responds to pathogens; and how it maintains ongoing protection after the disease using antibodies. Whilst there are many successful vaccines currently available, there are still no registered vaccines for some globally prevalent infectious diseases such as malaria and human immunodeficiency virus (HIV). Although we have made enormous progress in medicine over the last 300 years since the practice of vaccination first began, there are still diseases that are killing millions of people globally which desperately require a vaccine. Furthermore, there is a multitude of autoimmune conditions such as food allergies which may benefit from a traditional prophylactic vaccination approach. This review will explore the progression of traditional vaccines from empirical vaccines to the more recent novel vaccines and how recent advancements could change the field of allergy research.

## A BRIEF HISTORY OF VACCINATION

The first crude attempt at disease control was the procedure of variolation where the inoculated person stood a good chance at surviving both the procedure and later exposure to the pathogen. Variolation consisted of directly transferring the infection from a sick person to a healthy person, through direct contact or by infectious matter such as pus, saliva or blood (Dinc and Ulman, 2007). This form of vaccination is believed to have begun in either ancient China or India, but was only brought to the UK by the wife of a British diplomat, Lady Wortley-Montagu in 1721 (Dinc and Ulman, 2007).

Lady Wortley-Montagu had observed that harem girls in Constantinople had pox-free faces which were attributed to them being variolated; hence she had her son variolated in Istanbul in 1718 to save him from experiencing what she had as a young adult – smallpox. Later she also variolated her daughter in London; however, this was only after she had confirmed that it did not result in death or disease in eleven orphans and six convicted murderers from Newgate Prison (Dinc and Ulman, 2007). Lady Wortley-Montagu was so impressed that she implored her surgeon, Dr. Charles Maitland, to learn the technique and demonstrate it to the Royal British Court (Stewart and Devlin, 2006). After this demonstration, 200 upper-class members of British society, including members of the royal family, underwent the procedure, and in 1729 a further 897 more inoculations were performed with only 17 deaths post-procedure which is infinitely fewer than smallpox mortality at the time (Dinc and Ulman, 2007).

Even though Lady Wortley-Montagu was severely criticized for bringing the procedure to Britain, it was slowly implemented throughout the UK over the following years, and in 1757 a young boy named Edward Jenner would be variolated against smallpox (Dinc and Ulman, 2007). This ultimately saved countless lives from smallpox; the most devastating disease of the time. However, there were two issues with variolation: (1) it could impair the patient or even kill them if the dosage was incorrect or if they were not physically fit enough to withstand the infection, and (2) whilst the patient would be protected from further infections, they would become contagious during the active infection (Bazin, 2003).

Although variolation was popular in the cities, in the English countryside there were many rumors that if you contracted cowpox you were protected from the deadly smallpox. Subsequently, a farmer named Benjamin Jesty in Yetminster, England, inoculated his wife and two sons with cowpox in the hope of surviving a smallpox epidemic (Pead, 2003, 2006). Even though his wife became very ill, she and the whole family survived and went on to survive many smallpox epidemics in the area. This transpired a full 20 years before scientist Jenner began his experiment with a boy called James Phipps (Pead, 2003); however, Jesty was recognized for his contribution in 1805 by a published statement and a portrait commissioned by the Original Vaccine Pock Institution, London (Pead, 2006). It is believed that Jenner was also aware of the rumors of cowpox protecting against smallpox, and that this was the inspiration for his experiment, resulting in him being the first to document that a person infected with cowpox would survive subsequent exposure to smallpox (Stewart and Devlin, 2006). This technique evolved into using cow inoculums as the vaccine, which did provide immunity to smallpox although not to the same degree as natural disease or variolation. This discovery was heralded as the new age of vaccines and instigated new research into other common diseases.

A couple of centuries later, medicine would again make another considerable leap forward with the separate works of scientists Louis Pasteur and Robert Koch, and the publication of germ theory. The most famous of these works would be Pasteur’s and his attenuation of the bacteria *Pasteurella multocida,* which causes fowl cholera, by exposing the cultures to air and room temperature for extended periods of time (Bordenave, 2003). He demonstrated that whilst the bacteria were avirulent, they provided full protection from the virulent strain of the bacteria, which was a revolutionary idea at the time. Pasteur also went on to attenuate the rabies virus by passage through rabbits (Bazin, 2003). Koch, on the other hand, would discover the bacterial agents of anthrax, tuberculosis and cholera whilst also compiling postulates with fellow scientist Jacob Henle that would transform the world of microbiology (Kaufmann and Schaible, 2005). All of these discoveries led to the development of immunology and non-empirical vaccines.

The first whole cell vaccine was produced by Salmon and Smith in 1886 and was based on a *Salmonella* strain that was killed by heat and injected into pigeons to provide immunity (Bazin, 2003). Around the same time, others were investigating bacterial components and methods to purify them. This was the beginning of traditional vaccine methodology. During this era there were many great innovations in the field of immunology and vaccinology, such as the discovery of toxins and the consequent inactivation of toxins by heat and formalin, killed vaccines, adjuvants, sub-unit or acellular vaccines, tissue culture and live attenuated vaccines. With the establishment of molecular biology and genetic engineering in the late 1950s, a new era began where vaccine development no longer needed to be empirical and bacterial components could be produced artificially or even *in vivo* by unrelated vectors.

### VACCINES IN THE MODERN ERA

#### What makes a good vaccine?

The traditional definition of a vaccine is one that protects against a particular (or group of) infectious agent(s); however, these days there are many vaccines that could be designated as therapeutic agents against diseases such as cancer (Bergman et al., 2006), although the goal is still to prevent illness. In this review we will focus on prophylactic vaccines. The global market for vaccines is estimated to be around US$8 billion per year whilst the cost to develop each vaccine from concept to commercialism is around US$300–800 million (Plotkin, 2005). The reason for the high expenditure is that each vaccine has to be rigorously tested before commercial release and the average time it takes to fully develop a vaccine is between 15 and 20 years (Arntzen et al., 2005). A successful vaccine is measured by its effectiveness, its spectrum of protection, the duration of immunity and the strength of immunological memory that it induces. Secondary considerations of a good vaccine are its stability, ease of administration and storage, achievable mass production and its toxicity.

Biotechnology is a rapidly developing area which allows continued improvement into the exploration of antigens suitability as vaccine candidates. Choosing the right antigen is a core decision in the development of a vaccine candidate as some antigens that are immunogenic *in vivo* may not elicit long term protection. The same antigen may also vary in structure and sequence between strains, limiting its usefulness. Some antigens are also hard to express and purify on a large scale which is required for mass production (Mora et al., 2003). This is where novel vaccine methodology hopes to improve how vaccines are made and administered; this will be examined subsequently.

#### Routes of administration

The oldest technique for vaccination is that of subcutaneous delivery via scarification and one of the newest techniques is intramuscular injection (Bazin, 2003). Whilst the intramuscular route of vaccination is quite standard today in developed countries, it is an inconvenient method of application as it requires sterile needles and syringes, and usually a medical physician to administer it. This is the major drawback of vaccines that rely on intramuscular injections to be effective. In one study, a viral vectored vaccine was found to elicit stronger systemic and detectable mucosal responses via a single intramuscular injection than if it was applied via the oral route. The oral route proved to stimulate suboptimal T-cell responses and did induce a higher level of mucosal antibody than the intramuscular route (Lin et al., 2007).

Nasal and oral administration routes of a vaccine are more desirable than intramuscular as they are non-invasive, painless, not required to be sterile and do not require a physician for administration. This final point is most important as it is one of the reasons that third world countries have the lowest level of immunizations in the world (Costantino et al., 2007). Nasal immunization would place the vaccine in contact with the large surface of the nasal mucosa which consists of the nasal-associated lymphoid tissue (NALT), which can lead to both humoral and cellular immune responses (Zuercher et al., 2002; Costantino et al., 2007). The most well-known nasal vaccine is FluMist^®^; a live cold-adapted influenza virus. It can be given as one or two doses from a syringe sprayer, is licensed for use in the USA for persons aged 5–50 and has shown high efficacy from its inception (Plotkin, 2005; Costantino et al., 2007). However, one of the detriments of a nasal vaccine is that an unpleasant taste and nasal discomfort can occur often discouraging repeated use (Atmar et al., 2007).

Oral administration is a practical method of application if it can be achieved without diminishing the effectiveness of the vaccine, and immunity can be achieved with a single dose. The objective of oral vaccines is to mimic a natural infection and provide mucosal immunity. Orally delivered vaccines can induce suboptimal T-cell responses with high levels of mucosal antibody than the intramuscular route; however, the vaccine must be very stable as it will have to survive the acidic environment of the stomach before it reaches the M cells of the intestinal wall where it can be processed by antigen-presenting cells (APCs; Lin et al., 2007).

#### Adjuvants

Adjuvants are defined as compounds that influence the immune system into mounting a Th1 or Th2 response and whilst doing so, greatly enhances the magnitude of immune response against the antigen (Marciani, 2003). They are an important aspect of vaccines due to their tendency to make an ineffective antigen become effective. It is vital that adjuvants have the following properties: a non-toxic nature or have minimal toxicity at the dosage to elicit effective adjuvanticity; able to stimulate a strong humoral and/or T-cell immune response; provide good immunological memory or long-term protection; not induce autoimmunity; are non-mutagenic, non-carcinogenic, non-teratogenic, and non-pyrogenic; and be stable under broad ranges of storage time, temperature and pH levels (Marciani, 2003). The most popular adjuvants are aluminum-based and were first described and published in 1926 (Glenny et al., 1926). There are three adjuvants that are currently licensed for human use: aluminum hydroxide, also known as alum (Davies and Flower, 2007); monophosphoryl lipid A (MPL) and AS03 consisting of D,L-alpha-tocopherol (vitamin E), squalene and polysorbate 80 (U.S. Food and Drug Administration, 2014). It is well established that aluminum adjuvants stimulate the production of IgE and a Th2 immune profile, yet for some diseases this would not be adequate protection against pathogens as a Th1 response would be required (Lindblad, 2004). MPL and AS03 have demonstrated clinical efficacy when used in a HPV and influenza vaccine, respectively (Mohan et al., 2013).

Adjuvants are used to lengthen the dissemination time of the antigen from the site of injection which allows the antigen to be released over a prolonged period improving the effectiveness of the vaccine. This feature is called the depot effect and is traditionally associated with aluminum-based adjuvants (Lindblad, 2004; Mohan et al., 2013); however, recently the depot effect has been questioned as reducing the dissemination time of the antigen does not alter the magnitude of the immune response (Hutchison et al., 2012) along with other evidence suggesting other modes of action (reviewed in De De Gregorio et al., 2013). Other methods in which adjuvants improve the immune response are to form complexes with the antigen and to target the vaccine towards specific receptors. For example, the use of mannose in the adjuvant is recognized by pattern recognition receptors (PRRs) that initiate endocytosis and antigen processing (Stahl and Ezekowitz, 1998). The use of pathogen-associated molecular patterns (PAMPs) such as lipopolysaccharide (LPS) and CpG-DNA, and of synthetic low-MW imidazoquinolines in adjuvants, all trigger innate immune responses that lead to a Th1 or Th2 response in the vaccinated person (Marciani, 2003). Other adjuvants consist of cytokines (Cheng et al., 2007) and glycolipids (Singh et al., 1999; Ko et al., 2005) and other immunomodulators (Morrow et al., 2004) that bind to highly specific receptors on T cells which activate them.

### TRADITIONAL VACCINE METHODOLOGY

The early development of vaccines focused on using killed organisms, inactivated toxins or modified organisms, but currently there are many different approaches to vaccine development, which will be examined subsequently. As these approaches were empirical in design, these types of vaccines, whilst being successful, are now viewed as being traditional vaccines. These can be divided into three different types: (a) killed vaccines; (b) attenuated vaccines; and (c) sub-unit vaccines.

#### Killed vaccines

A killed or “inactivated” vaccine is developed by the pathogen being grown and then being made inactive by means of heat, chemical or radiation treatment and was the basis of most vaccines until the 1980s. This results in the pathogen being unable to cause disease whilst providing the immune system with stimulation via its normal antigenic epitopes on its cell surface. One major disadvantage of this approach is that, whilst these vaccines are immunogenic, they do not replicate *in vivo* infectivity limiting the spectrum of the immunity acquired as the agent is incapable of going through its normal antigenic variation over the course of the infection. This results in decreased immunity and a requirement of booster shots to maintain immunity (Moylett and Hanson, 2003). Another disadvantage of this vaccine type is that during the inactivation process the antigenic epitopes can be modified resulting in a less efficacious vaccine (Tano et al., 2007). Despite these limitations, killed vaccines are commonly used today with the typhoid, Salk poliomyelitis, and seasonal influenza vaccines still being administered (Bazin, 2003; Palese, 2006).

#### Attenuated vaccines

Among the more efficacious of the traditional vaccines are the attenuated ones. In this case, a pathogen is subjected to altered growth conditions, is passage through a host or is genetically modified to eliminate its virulence, yet retaining its ability to replicate albeit at a greatly reduced rate. These vaccines are more successful at eliciting a robust lifelong immunity than other traditional vaccines. This can be attributed to their ability to cause an asymptomatic infection which stimulates both the humoral and cellular branches of the immune system.

However, this ability to replicate carries the greatest risk as the vaccine can persist in immune-compromised persons or the elderly due to limited immune responses. A benefit to these vaccines is they express their own immunogenic antigens which stimulate the immune system strongly thereby negating the need for an adjuvant to be used (Loessner et al., 2008). The most commonly used attenuated vaccine is the MMR vaccine which protects children worldwide against measles, mumps and rubella and with subsequent boosters provides lifelong immunity (Vandermeulen et al., 2007). Attenuated vaccines have further progressed into carrier vaccines where they can deliver heterologous antigens (Bachtiar et al., 2003; Lotter et al., 2008; Schoen et al., 2008). For live carrier vaccines that deliver multiple heterologous antigens, there is a risk that the host immune system will dampen the immune response to the heterologous antigens by misdirecting the immune response against the carrier (Berzofsky et al., 2004). However, if the immunity induced is cell-mediated the response can be enhanced by pre-existing immunity to the carrier strain (Saxena et al., 2013).

#### Subunit vaccines

Traditionally, it was thought that the only way to protect against a disease was to use the whole organism to vaccinate the host. However, it was elucidated that specific parts of the organisms, when purified or isolated, demonstrated immunogenic properties. These components could be the capsule, the flagella or even an outer membrane protein of the cell wall. These types of vaccines are known as subunit vaccines or acellular vaccines. These vaccines are not able to cause the disease and in comparison to whole cell killed vaccines they are not as efficacious. This is both an advantage as they are safe for immune-compromised patients and a disadvantage as they do not elicit long-term immunity and will often require multiple vaccinations to maintain immunity (Schmitt et al., 2008). An advantage of this type of vaccine is that it can be engineered to protect against various strains of the organism. An example of a successful subunit vaccine is the *Haemophilus influenzae* type b (HiB) conjugate vaccine which consists of a polysaccharide-protein conjugate. This vaccine has eliminated or significantly reduced this disease in children in regions of South America (Ribeiro et al., 2007; Franco-Paredes et al., 2008) and Africa (Adegbola et al., 2005; Muganga et al., 2007) where it was once endemic. In the UK, the success of this vaccination program was compromised by a highly publicized paper (which has now been retracted) that linked autism to early childhood vaccination which lead to a rise in HiB infections as parents chose not to vaccinate; however, subsequent booster campaigns by the NHS has seen a reduction in infection rates again (Ladhani et al., 2008). A recent meta-analysis covering studies involving over 1.2 million children has discredited any link between vaccinations or vaccine components thimerosal or mercury to the development of autism or autism spectrum disorders (Taylor et al., 2014).

### DNA VACCINES – A NEXT GENERATION EXAMPLE

There are multiple novel types of vaccines that are currently under development, such as bacterial ghosts (Szostak et al., 1996; Jawale and Lee, 2014) and nanovaccines (Cho et al., 2014). However, one that holds great promise and has had documented successes is DNA vaccines. DNA vaccines differ from traditional vaccines as they do not consist of a protein or a cell component but only the DNA that encodes an immunogenic antigen within a plasmid vector. The plasmid can be administered by injection, gene gun, electroporation, or aerosol delivery, upon which the host’s immune cells, usually dendritic cells, will sample the plasmid and express the encoded antigens. These antigens are then degraded by the cell into peptides and presented via MHC class I and class II molecules depending on the mode of administration and the cell type. From this, both antibody and cellular responses can be induced (Forde, 2005).

The first reported use of a plasmid DNA vaccine outside of trial or experimental conditions was in 2003 and was a desperate attempt to save an endangered species from extinction (Bouchie, 2003). The vaccine was for the highly endangered California condors against the lethal West Nile virus. West Nile virus had emerged in New York in 1999 and spread to 41 out of the 50 US states killing birds from 138 species in a matter of years. It was believed that if the virus spread to California, the remaining 200 or so condors would face extinction. The US Centers for Disease Control and Prevention (CDC) expedited the manufacture of an experimental vaccine and permitted the condors to be vaccinated with it (Bouchie, 2003). The DNA vaccine expressed West Nile virus pre-membrane/membrane and envelope proteins. The vaccinated condors were monitored and it was observed that the DNA vaccination stimulated protective immunity in adults, nestlings and newly hatched chicks. Following two intramuscular vaccinations, the condors demonstrated excellent neutralizing antibodies 60 days post-vaccination with a continued increase until approximately 1 year post-vaccination. It was also noted that the birds did not show any unusual behaviors, health changes or side effects post-vaccination (Chang et al., 2007). This vaccine has also demonstrated efficacy in other bird species such as the American robins (*Turdus migratorius*) (Kilpatrick et al., 2010) and the fish crows (*Corvus ossifragus*; Turell et al., 2003). The first two DNA vaccines for veterinary use were granted US approval in 2005 for West Nile virus vaccine for horses and haematopoietic necrosis vaccine for farm-reared Atlantic salmon (Chalmers, 2006). Even though there are no currently approved DNA vaccines for human use, as of May, 2014 there are 128 open trials listed on Clinicaltrials.Gov (2014) that involve DNA-based vaccines and therapies

### GLOBAL VACCINE SUCCESS

The global eradication of smallpox is, to date; the most successful vaccination campaign in history. Smallpox has existed for many thousands of years and spread through the world following the migration of humans to new settlements (Barquet and Domingo, 1997). As mentioned previously, Edward Jenner is famously credited with developing a smallpox vaccination using the cow-pox virus (vaccinia virus) and published many observations on both the successful and adverse events (Jenner, 1809) associated with his vaccination protocol. Small pox was an indiscriminate disease that is caused by two virus variants Variola major and Variola minor and was responsible for 300–500 million deaths before its eradication (Theves et al., 2014). The smallpox vaccine that was developed by Jenner produces both neutralizing antibodies and cell mediated responses that are protective against other members of the Orthopoxvirus genus (Barquet and Domingo, 1997). After years of vaccination success but with deaths from smallpox still common, the World Health Assembly, the executive body of the [World Health Organization (WHO), 2013] set a target to eradicate smallpox. This was only achievable as humans are the only reservoir for the virus and the vaccine had demonstrated high efficacy (Fenner et al., 1988). In the late 1960s, the efforts of the WHO were strengthened with more funding and new surveillance protocols.

The last natural occurrence of smallpox occurred in Somalia, where cook Ali Maow Maalin developed the rash on October 26th 1977, but tragically it was not the last global smallpox death [World Health Organization (WHO), 1980]. Medical photographer Janet Parker became the last person to die of smallpox in the world when she was accidently exposed to it in her workplace at the University of Birmingham and unfortunately a lapse in obtaining her booster vaccination led to her being susceptible at the time of exposure (Barquet and Domingo, 1997). Eradication of smallpox was declared on May 8, 1980 by the WHO when the Final Report of Global Commission for Certification of Smallpox Eradication was published [World Health Organization (WHO), 1980]. As of 2014, two depositories of smallpox still exist at the CDC in the USA and the State Research Center of Virology and Biotechnology VECTOR in Koltsovo, Russia. The destruction of these viral stocks has been delayed and debated since the declaration of eradication occurred in, 1980. Discovery of smallpox victims during building excavations often fuels these debates although no viable virus has been recovered from these corpses, so the risk of a modern smallpox outbreak is improbable (Reardon, 2014; Theves et al., 2014). The WHO is again debating the existence of these stocks in May, 2014 (Reardon, 2014).

Another successful vaccine that has been implemented globally is those against poliomyelitis – the Salk, and Sabin vaccines. There are three different poliovirus serotypes and all of them can lead to serious disability in children, even death by acute flaccid paralysis [World Health Organization (WHO), 2014a]. Due to its moderate mortality rates, its long-term severe disability consequences and like smallpox, humans being the only natural reservoir for the virus, the World Health Assembly set a target of eradication by the year 2000. This project is known as the Global Polio Eradication Initiative. Poliovirus Type 2 infection has not been observed since 1999 in India and Type 3 since 2012. However, in 2014, poliovirus Type 1 is still endemic in regions of Nigeria, Pakistan, and Afghanistan [World Health Organization (WHO), 2014a]. The reasons behind these persisting endemics will be discussed later.

There are two vaccines, an oral live attenuated vaccine known as the Sabin vaccine and the inactivated poliovirus vaccine also known as the Salk vaccine [World Health Organization (WHO), 2014a]. The Sabin vaccine was derived from passages of the poliovirus strains through rats and mice and then through cell cultures more than 50 times resulting in an attenuated forms of the virus types that all induced good antibody levels (Sabin, 1957; Baicus, 2012). In 1972, Sabin donated his vaccine strains to the WHO which increased the number of vaccine recipients from 5 to 80% [Baicus, 2012; World Health Organization (WHO), 2014a]. The Sabin vaccine is no longer in use in the USA or UK as the only poliomyelitis cases reported in the populations were vaccine-associated paralytic poliomyelitis where the vaccine strain has caused an outbreak but it is still used in some developing countries due to its ease of administration and cost (US$0.14 a dose vs US$2–3 a dose for Salk vaccine; Willyard, 2014). There are now plans to eliminate the Sabin vaccine entirely in the 124 countries that still use it by 2015 (Willyard, 2014).

The Salk vaccine is grown in monkey kidney cells and inactivated with formalin (Salk et al., 1954) and was introduced in the USA in 1955 and by 1961, the incidence of poliomyelitis had decreased from 13.9 cases per 100,000 in 1954 to 0.8 cases per 100,000 in 1961 [Baicus, 2012; World Health Organization (WHO), 2014a]. Besides preventing deaths, the main benefit to come from polio vaccination is the cost savings to the healthcare system which is estimated at US$40–50 billion for the period between 1988 and 2035 in the USA alone [World Health Organization (WHO), 2014a]. Most countries that have been certified polio-free still have rare isolated cases which have come from travelers importing the virus from endemic areas, for example in Australia had one such case in 2007 (Paterson and Durrheim, 2013). However, the Global Polio Eradication Initiative has a new timeline for eradication and with a new strategy of phasing out the Sabin vaccines, hopefully the world will be certified polio-free in 2018 (Willyard, 2014).

A more recent vaccine accomplishment is the pneumococcal conjugate vaccine (PCV) against *Streptococcus pneumoniae* (pneumococcus) infections which include acute otitis media, sinusitis, pneumonia and invasive pneumococcal diseases such as meningitis and sepsis. The first conjugate vaccine was a heptavalent vaccine which protects against seven different serotypes of pneumococcus and it was licensed in the USA in 2000 (Black et al., 2000; Lee et al., 2014). Since that time, 10- (Domingues et al., 2014), 13- (Spijkerman et al., 2013), and 23-valent (Grabenstein and Manoff, 2012) vaccines have been licensed with all producing strong immunity against a broad spectrum of strains. In the USA, all age groups from children under 5 years to adults over 65 years had dramatic reductions in incidence of pneumococcal infections over a seven year period after the PCV was available (Pilishvili et al., 2010).

It is predicted that if the heptavalent PCV was implemented in China it would prevent 4222 cases of invasive pneumococcal disease, 4,061,524 cases of otitis media and 472,527 cases of pneumonia, as well as preventing an additional 2682 deaths from pneumococcal disease; however, the implementation cost would be estimated at US$6.44 billion (Che et al., 2014). The current overall cost of pneumococcal disease in the unvaccinated population in China is estimated to be US$3.5 billion (Che et al., 2014). Following the introduction of PCV in the USA, an estimated 211,000 serious pneumococcal infections and 13,000 deaths were prevented in the period of 2000–2008 (Pilishvili et al., 2010). The influence of this vaccine on public health is in its early stages and has already had impacts on child mortality in over 88 countries that have included various PCV on their recommended immunization schedule (Whitney et al., 2014).

There are other vaccines that have been successfully implemented in the past decade. The most recent and highly publicized vaccine is the quadrivalent human papillomavirus vaccine against cervical cancer, marketed as Gardasil^®^, which prevents the premalignant disease that leads to cervical cancers and fulfills all the above criteria of being a successful vaccine (Zhou et al., 1991; Govan, 2008). Initially the cost of Gardasil^®^ was extremely prohibitive at US$120 per dose with three doses required; however, in collaboration with GAVI Alliance, the cost from the supplier has dropped to US4.50 per dose which increases its affordability and likelihood of being implemented in developing countries (Anon, 2013). As the cost of the vaccine decreases and more people are immunized this vaccine which has been included in over 30 countries immunization schedules, in conjunction with regular Pap screening, may lead to a long-term reduction in cervical cancer incidence (Harper et al., 2010; Ribeiro-Muller and Muller, 2014).

### VACCINE FAILURES AND CHALLENGES

Historically there have been more vaccine failures than successes and unfortunately those failures can be publicized and instill fear in the general public long after the event. One such failure is one that occurred early in the rollout of the Salk polio vaccine is known as the Cutter incident. In April, 1955 a few weeks after Salk’s polio vaccine had been declared safe and efficacious, there were reports from California that five children had become paralyzed after receiving the vaccine (Offit, 2005). These vaccines were traced to Cutter which was one of the five pharmaceutical companies that were granted a license to produce the vaccine in the USA (Nathanson and Langmuir, 1995). It was found that two production batches failed the deactivation steps; so live virulent poliovirus was found in 120,000 doses of the vaccine. Of the children vaccinated from this pool, 40,000 developed abortive polio, 51 suffered from permanent paralysis and five died (Nathanson and Langmuir, 1995). Unfortunately this was not the end of the tragedy, a polio outbreak followed where a further 113 people in close contact with the vaccinated children were infected and subsequently paralyzed, and a further five deaths (Nathanson and Langmuir, 1995; Offit, 2005). This incident halted the implementation of the polio vaccine program and severely affected public confidence in the vaccination, not only in the USA but as far reaching as New Zealand (Day, 2009), Germany, the UK and Sweden (Axelsson, 2012) and in the end, it caused the USA to recommend Sabin’s vaccine in the long term which, barring manufacturing failures, proved to be the more risky of the two vaccines as it could revert to full virulence and cause outbreaks of vaccine-associated paralytic poliomyelitis (Offit, 2005; Fitzpatrick, 2006).

Following its emergence in 1981, HIV infections and its subsequent disease acquired immunodeficiency syndrome (AIDS) has become a global pandemic with millions of deaths and over 34 million people living with HIV (De Cock et al., 2012). According to the [World Health Organization (WHO, 2014c) ] and the Joint United Nations Programme on Hiv/Aids (2013) the pandemic appears to have peaked as AIDS-related deaths have decreased by 25% in the past decade as well as new infections decreasing by 20% since 2006. This is the combined effect of the development of anti-retroviral drugs, and better education about the transmission of this disease. However, a vaccine is desperately needed to prevent new infections and to stop this pandemic from affecting future generations.

Multiple HIV vaccines have been tested in clinical trials with limited success (Johnson et al., 2013). In the last decade, the most prominent vaccine trial failures was that of the Merck STEP phase II test of concept and efficacy trial for an Adenovirus5 (Ad5) vaccine. It showed that the MRKAd5 HIV-1 gag/pol/nef vaccine was highly immunogenic and elicited a higher magnitude of HIV-specific CD8+ T cells than any of the other HIV candidate vaccines over the past 15 years but it did not prevent HIV infection or reduce viral loads in infected patients (Buchbinder et al., 2008). In fact, more disturbingly, there was an increase in the number of HIV-1 infections in male recipients of the vaccine compared to the controls (McElrath et al., 2008). This trial was immediately ceased when the independent data and safety monitoring board determined that the study could not demonstrate efficacy (Buchbinder et al., 2008).

One of the reasons behind the failure of the Merck STEP clinical trial was the pre-existing neutralizing antibodies against Ad5. A recent study confirmed that the international epidemiology of pre-existing immunity to different adenovirus types can severely compromise its efficacy as only 14.8% of the 1904 participants were seronegative for neutralizing antibodies against Ad5 (Mast et al., 2010). This indicates that naturally acquired infections from virulent forms of the vaccine vectors can limit their usefulness in the same species. However, choosing a virus from a different species for which no prior exposure is possible but may sound too risky to be accepted by the general population. It was also found that whilst the group of men that became more susceptible to HIV infection post-vaccination were seropositive against the Ad5 vector, they were also uncircumcised and had sexual relations with the same sex implying that pre-existing immunity may not be the sole factor that caused this vaccine failure (Gray et al., 2010; Duerr et al., 2012). Whilst this phase II trial failure was a major setback for the HIV research community, it raised fundamental questions about the pathogenesis of HIV and also gave insight into immunological mechanisms that were previously unexplored (Johnson et al., 2013; Fauci et al., 2014). The search for a HIV vaccine is ongoing and as of May 2014, there are 92 open HIV vaccine trials according to Clinicaltrials.Gov (2014).

Another infectious disease that is under surveillance by health departments worldwide is a double-stranded RNA virus called rotavirus. Rotavirus causes acute enteritis resulting in severe, dehydrating diarrhea in infants and young children and is very transmissible through close contact [Bishop et al., 1976; World Health Organization (WHO), 2013]. In the pre-vaccination era, rotavirus caused 111 million cases of illness with 25 million medical visits, 2 million hospitalizations and between 352,000 and 592,000 rotavirus gastroenteritis-associated deaths worldwide annually with most of these occurring in low income countries (Parashar et al., 2003). The first rotavirus vaccine was RotaShield which was developed by Wyeth-Lederle Vaccines and Pediatrics, Philadelphia, as an oral vaccine and showed high efficacy at 80% protection from severe illness; hence it was recommended for all infants in the USA once it was approved by the Food and Drug Administration (FDA) on August 31, 1998 (American Academy of Pediatrics, 1998). Over the eleven month period after the vaccine was approved until July 7, 1999, 15 cases of intussusception, a type of intestinal blockage requiring surgical intervention, were reported and linked to the vaccination. In consultation with the FDA, Wyeth-Lederle Vaccines withdrew Rotashield from the market on October 15, 1999. Before this withdrawal, the cases of confirmed intussusception had risen to 101 (Delage, 2000) and fortuitously, because there were no deaths caused by this vaccine, physician trust in vaccine safety measures were not compromised by this withdrawal (McPhillips et al., 2001).

In 2006, two new oral rotavirus vaccines were released onto the market: Rotarix^®^ – a live monovalent attenuated human strain by GlaxoSmithKline Biologicals (Vesikari et al., 2004; Keating, 2006b) and RotaTeq^®^ – a live pentavalent human-bovine reassortant vaccine by Merck & Co. Inc. (Clark et al., 2004; Keating, 2006a). After 6 years of use, a Cochrane Review found that both of these vaccines are efficacious with no increased risk of adverse side effects such as intussusception (Soares-Weiser et al., 2012). However, in 2013, a small increase in risk was confirmed when the data was analyzed comparing the risk of intussusception in the post-vaccine period with other periods (Haber et al., 2013; Quinn et al., 2014).

A year later, the vaccines are still on the market albeit with an intussusception warning even though there is an estimated up to sixfold increase with the use of these two rotavirus vaccines. So far the Vaccine Safety Datalink has reported that Rotarix^®^ has had 66 intussusception cases in 200,000 doses, whilst RotaTeq^®^ had eight cases for 1.3 million doses administered with most occurring within 7 days after the first dose [World Health Organization (WHO), 2014b]. Currently the risk of intussusception is estimated to be 1–2 per 100,000 infants vaccinated [World Health Organization (WHO), 2013]. However, the general view is that there are great benefits to vaccination against rotavirus as the infant mortality rates in countries that have added this to their vaccination schedule have significantly decreased (Buttery et al., 2014) and this is reflected in the WHO’s Global Advisory Committee on Vaccine Safety in their weekly epidemiological record [World Health Organization (WHO), 2014b] stated this in regards to the new intussusception risk: “the findings remain reassuring that the risk of intussusception following current rotavirus vaccines remains small compared to the benefits of preventing the impact of severe diarrhea.” Surveillance of such adverse effects requires long-term study in order to make sound decisions about the appropriateness of the vaccine. There may come a time where the relative risk is too high and the vaccine is withdrawn like Rotashield which had a rate of intussusception of 1 in 10,000 infant doses [World Health Organization (WHO), 2013], even though it provided strong immunological protection. This is one of the hardest aspects in vaccine development to plan for and may lead to public distrust in future vaccines, if it is not done expediently when those risks increases.

### POLITICAL AND GLOBAL ASPECTS OF VACCINE USAGE

When a vaccine is designed, it is assumed that if it proves effective it will be used in various countries around the world to vaccinate the population; however, this is not always the case. Within each country there are government agencies, industry and community health advocates, and outside agencies such as the WHO that will make recommendations for vaccination strategies. Often this process will result in a successful vaccination strategy such as the global eradication of smallpox (Stewart and Devlin, 2006), but it can also lead to confusion and scepticism in the chosen strategy. One such example was the choice of pertussis vaccine for a national vaccination campaign in the Netherlands.

Originally, the Dutch government chose to use a whole cell vaccine based on the *Bordetella pertussis* bacterium; however, after speculation that the vaccine could cause brain damage, alternative vaccines were sought. At this time, acellular vaccines comprising three to five bacterial components were being used by many countries in Europe as they were comparable in protection to the whole cell vaccines and demonstrated minimal side effects (Blume and Zanders, 2006). Over the course of 7 years, the debate over the new vaccine became very convoluted as many government agencies, drug companies, and consumer groups presented opposing studies and evidence. There was also external pressure from neighboring countries and global non-profit groups including the WHO and United Nations Children’s Fund (UNICEF) for the Dutch government to make a decision. Concurrently, many parents had lost faith in the old vaccine strategy; hence an epidemic of pertussis ensued. To combat the growing epidemic the Dutch government chose an acellular vaccine which was used in primary vaccinations in 2005; however, the Health Minister advised that this decision was not based on recommendations and evidence provided by the Dutch Health Council, but on the need to appease parents and re-establish their confidence in the vaccine strategy (Blume and Zanders, 2006). By contrast, in areas where any disease is endemic and the health system is overwhelmed, often the vaccination strategy proposed by governing bodies will be accepted by the population and acquiesced as mandatory (Chalmers, 2006).

Unfortunately this has not worked in areas such as Pakistan, Nigeria, and Afghanistan where the eradication of polio has failed due in part to misinformation, violence, politics, and mistrust about vaccination. There is a distinct divide in these populations between vaccine acceptors and non-acceptors which is based in the abundance of misinformation about the vaccine, religious beliefs and the emotional fear about the agenda; however, if there is an outbreak many non-acceptors will accept the vaccination as the fear of disease outweighs the perceived risks (Murele et al., 2014). Socio-cultural, educational and perceptual factors are particularly strong in these regions and in some cases targeting male authority figures could improve vaccination uptake (Murele et al., 2014); however, in other regions maternal education and empowerment has been suggested as a strong motivator in vaccine acceptance (Larson et al., 2014).

Violence is another contributing factor to this program’s failure particularly when there are fatal attacks on vaccination workers in Pakistan and Nigeria (Abimbola et al., 2013). In Afghanistan, both the Taliban regime and the militant Islamist terrorist group Al Qaeda support the Global Polio Eradication Initiative; however, factions within these groups can disrupt it as they view it as a Westernization issue, rather than a health one (Abimbola et al., 2013). In Nigeria and Pakistan, militants can gain international media attention by attacking polio health workers (Riaz and Rehman, 2013) and spreading propaganda that immunization programs are actually covert sterilization campaigns to reduce the Muslim population, which puts more fear into the local communities than the disease itself (Abimbola et al., 2013; Willyard, 2014).

All of the aforementioned issues affect the successful eradication of infectious diseases with well documented epidemiology and pathology. However, there exist conditions and disorders where the mechanisms of development and ongoing chronic pathology are yet to be fully ascertained. One such condition causing concern among health professionals globally is allergy.

## ALLERGY AND VACCINE POTENTIAL

Allergy is a hypersensitivity disease characterized by the production of IgE antibodies against antigenic components (i.e., allergens) that can enter the body via the respiratory and gastrointestinal tract, the skin, an insect sting or injection of a drug (Sicherer and Sampson, 2014). The clinical reactions experienced by sensitized patients vary in different target organs and include rhinitis, urticaria, and allergic asthma to life-threatening anaphylactic shock (Sampson, 2003, 2004). The acute symptoms of allergy are usually due to the release of inflammatory mediators by tissue-bound mast cells and circulation basophils. These inflammatory mediators include histamine, platelet-activating factor, leukotrienes, mast cell proteases, and a range of cytokines. Mediators are released when allergen binds to IgE antibody attached to FεRI receptors on the cell surface, causing degranulation. Studies show a skewing towards a Th2 response, with elevated levels of IL-4, IL-5, and IL-13, while tolerant individuals usually have higher levels of the Th1 cytokines IFN-gamma and TNF-alpha, and the regulatory cytokine IL-10 (Andre et al., 1996; Noma et al., 1996; Schade et al., 2003; Turcanu et al., 2003; Tiemessen et al., 2004). The class switch to produce IgE antibody occurs during primary sensitization in allergic patients and seems to be driven by IL-4, which is a direct product of Th2 cells and other effector cells of the allergic immune response. The activation of allergen-specific T cells is achieved by the presentation of allergens via APCs, including dendritic cells (Grainger et al., 2014; Nagai et al., 2014).

As the prevalence and potential fatality of this disease has increased, so have the efforts to find effective therapies and prophylaxis also intensified (Valenta et al., 2010). Specific immunotherapy (SIT) is effective for desensitization against inhalant allergens; however, it is not advised as a therapy against food allergy because of the high risk of adverse side-effects (Sabato et al., 2014). Oral administration of antigens usually leads to tolerance, and has been effective in decreasing allergic sensitization to antibiotics and other medications (Stevenson, 2000, 2003). Obviously native food allergens cannot be administered in this way, but it may be possible for hypoallergenic or CpG-conjugated derivatives. Microencapsulation provides a promising way of delivering allergens without degradation in the stomach (Litwin et al., 1996), thereby inducing oral tolerance, and has already been applied in clinical trials (TePas et al., 2004). Conjugation or co-administration of recombinant allergens with Th1-inducing heat-killed bacteria has yielded good protective results in mice (Li et al., 2003a,b) and allergic dogs (Frick et al., 2005). Various approaches have been attempted to develop safe and effective DNA vaccines and are discussed in the following section.

### DNA VACCINES AND ALLERGY

DNA vaccines, as demonstrated in the California condors, can induce protective immune responses against infectious diseases. Plasmid DNA injected intramuscularly, intraperitoneally or with a gene gun results in transcription and translation of encoded genes and elicits an antibody response in the host (Tang et al., 1992; Ulmer et al., 1993; Hsu et al., 1996b). This method of immunization preferentially induces a Th1 immune response and suppression of IgE (Raz et al., 1996; Yoshida et al., 2000). These effects appear to be mediated by both CD8+ and CD4+ cells (Hsu et al., 1996a; Lee et al., 1997; Peng et al., 2002), and plasmid DNA requires immunostimulatory sequences such as CpG for optimal immunogenicity (Sato et al., 1996; Adel-Patient et al., 2001; Jilek et al., 2001; Hartl et al., 2004). Unmethylated CpG motifs either in bacterial DNA or as synthetic oligodeoxynucleotides (CpG-ODN) are recognized by the mammalian immune system via toll-like receptor 9 (and possibly other PRRs) and trigger a Th1 response (Hartmann and Krieg, 1999; Stacey et al., 2000; Bauer et al., 2001). Experiments in murine models of allergic asthma, rhino sinusitis, and conjunctivitis show that administration of CpG-ODN alone prevents symptoms and reduces already established disease by reducing Th2 immune responses and IgE (Kline et al., 1998, 1999; Magone et al., 2000; Serebrisky et al., 2000). Allergen/CpG-ODN conjugates have been shown to be less allergenic and more immunogenic than native allergen (Tighe et al., 2000; Horner et al., 2002). The major allergen from ragweed, Amb a 1, linked to an immunostimulatory DNA sequence promoted Th1 cytokine expression and down regulated Th2 expression *in vitro* (Simons et al., 2004), reversed established airway hyperreactivity in a murine model of asthma (Marshall et al., 2001; Santeliz et al., 2002) and yielded promising results in Phase II clinical trials (Tulic et al., 2004).

Genetic immunization to specific allergens using plasmid DNA offers a powerful solution to the major problems associated with protein immunization, such as cross-linking of IgE antibody on effector cells or even *de novo* synthesis of IgE antibodies to the immunized protein itself. However, genetic vaccination may lead to an uncontrolled synthesis of allergens in the vaccinated host (Slater et al., 1998) and has been a major hurdle for application in allergic patients. Three approaches are currently used to prevent this from occurring: (a) cutting the allergen-coding gene into fragments, lacking the antigenic determinant but containing the original T cell epitope repertoire, (b) the use of hypoallergenic protein derivatives, or (c) fusing allergen with proteins that promote immune responses.

Several allergens have been tested in DNA vaccination approaches using murine models, including Ara h 2 (peanut), bovine beta-lactoglobulin (cow’s milk), Cry j 1 (Japanese cedar), phospholipase A2 (bee venom), Der f 11 and Der p 1 (dust-mite), and Bet v 1 and Phl p 2 (grass; Roy et al., 1999; Toda et al., 2000; Adel-Patient et al., 2001; Jilek et al., 2001; Kwon et al., 2001; Peng et al., 2002; Hochreiter et al., 2003; Ludwig-Portugall et al., 2004). Most studies observed elicitation of a Th1 response and increased IL-10 production. Mice vaccinated against phospholipase A2 were protected against fatal anaphylaxis following allergen challenge (Jilek et al., 2001), while mice receiving an oral DNA vaccine containing the peanut allergen Ara h 2 (Roy et al., 1999) experienced significantly less severe and delayed allergic reactions upon subsequent sensitization and challenge. However, prophylactic effects, while promising, are not sufficient to aid patients who have existing food allergy. In mice pre-sensitized to phospholipase A (bee venom), therapeutic gene vaccination prevented only 30% of mice from anaphylaxis (Jilek et al., 2001).

In addition to direct DNA vaccination, these approaches provide the option of co-delivering genes or adjuvant molecules with immunomodulatory properties together with the antigen sequence (Hartl et al., 2004; Mutschlechner et al., 2009). Allergen–allergen hybrid molecules may combine different allergens from one complex allergen source or use allergens from different sources as demonstrated for grass pollen (Linhart et al., 2005; Wallner et al., 2009). Furthermore, hybrid molecules using only T cell epitopes have been successfully used (Linhart et al., 2008). Vaccination of mice with a plasmid containing the cDNA for OVA fused to the cDNA of IL-18 (Allergen–cytokine fusion protein), a potent Th1 inducer, reversed established airway hyperreactivity, while a plasmid containing OVA alone had only a prophylactic effect (Maecker et al., 2001).

Ubiquitination of allergens represents another routine approach for destroying IgE-binding epitopes on proteins to produce hypoallergenic DNA vaccines. This approach has been applied for the production of a DNA-based vaccine encoding an ubiquitinated version of *Linhart* v 1, the major allergen from birch pollen (Bauer et al., 2006). It was demonstrated in a murine study that this vaccine did not produce any detectable antibody response, but T cell reactivity was preserved as well as allergic reactions prevented.

In summary, several novel therapeutic and prophylactic therapies against allergy are currently under investigation (Nieuwenhuizen and Lopata, 2005; Flicker et al., 2013; Weiss et al., 2013). Genetic immunization has proven a powerful method to induce anti-allergic immune responses. The underlying functional principle described seems to be based on the recruitment of allergen-specific Th1 cells, CD8+ cells and the establishment of a Th1 cytokine milieu. This response can be protective by preventing the development of a Th2-biased response towards allergens, as well as balance an ongoing Th2-type response in a more therapeutic application. More studies are needed to increase our understanding of the pathophysiology and immunological mechanisms of allergy, and to characterize the molecular structure and epitopes of allergens, to develop safer and more effective ways of combating this debilitating and potentially life-threatening disease.

## CONCLUSION

The advent of vaccination changed global society and our everyday lives dramatically, especially in conjunction with improved healthcare, infrastructure and technology. Over the last century with increasing knowledge of the immune system and infectious diseases, infant mortality associated with infectious diseases dropped, in developed countries debilitating illnesses like polio disappeared from public view, and the youth of today did not experience the threat or fear of death via infectious diseases. However, some diseases such as HIV and malaria are yet to have efficacious vaccines developed and successfully complete Phase III clinical trials. So the fight continues against these known enemies and with each failure, we learn more. The list of global health threats consists of many incurable infectious diseases; immunological disorders such as allergy should be added to that list. Currently, therapeutic interventions are adequate, but with population and allergy prevalence increasing there is a strong need for a prophylactic vaccine. Although the establishment of allergy is not fully elucidated, researchers should be mining the already long history of infectious disease vaccines to create new avenues of allergen vaccine development.

## Conflict of Interest Statement

The authors declare that the research was conducted in the absence of any commercial or financial relationships that could be construed as a potential conflict of interest.
